# Framework of Intrinsic Immune Landscape of Dormant Prostate Cancer

**DOI:** 10.3390/cells11091550

**Published:** 2022-05-05

**Authors:** Nelson K. Y. Wong, Xin Dong, Yen-Yi Lin, Hui Xue, Rebecca Wu, Dong Lin, Colin Collins, Yuzhuo Wang

**Affiliations:** 1 Department of Experimental Therapeutics, BC Cancer, 675 W 10th Ave, Vancouver, BC V5Z 1L3 Canada; nwong@bccrc.ca (N.K.Y.W.); xdong@bccrc.ca (X.D.); hxue@bccrc.ca (H.X.); rwu@bccrc.ca (R.W.); dlin@bccrc.ca (D.L.); 2 Vancouver Prostate Centre, Department of Urologic Sciences, Faculty of Medicine, University of British Columbia, 2660 Oak Street, Vancouver, BC V6H 3Z6, Canada; yen-yi.lin@ubc.ca (Y.-Y.L.); ccollins@prostatecentre.com (C.C.)

**Keywords:** prostate cancer, dormancy, androgen-deprivation therapy, immune surveillance, immune evasion, immunomodulation

## Abstract

Androgen deprivation therapy (ADT) is the standard therapy for men with advanced prostate cancer (PCa). PCa often responds to ADT and enters a dormancy period, which can be recognized clinically as a minimal residual disease. However, the majority of these patients will eventually experience a relapse in the form of castration-resistant PCa with poor survival. Therefore, ADT-induced dormancy is a unique time window for treatment that can provide a cure. The study of this well-recognized phase of prostate cancer progression is largely hindered by the scarcity of appropriate clinical tissue and clinically relevant preclinical models. Here, we report the utility of unique and clinically relevant patient-derived xenograft models in the study of the intrinsic immune landscape of dormant PCa. Using data from RNA sequencing, we have reconstructed the immune evasion mechanisms that can be utilized by dormant PCa cells. Since dormant PCa cells need to evade the host immune surveillance for survival, our results provide a framework for further study and for devising immunomodulatory mechanisms that can eliminate dormant PCa cells.

## 1. Introduction

Cancer recurrence is a major clinical problem. After effective intervention, the cancer patients may carry minimal residue diseases, either detectable or even undetectable, for a long period of time. However, cancer recurrence can occur in some of these patients as loco-regional lesions or distant metastases. This latency period between effective clinical intervention and cancer recurrence can be explained by cancer dormancy, which is categorized as tumor mass dormancy or cellular dormancy [1]. When a tumor enters tumor mass dormancy, the generation and death of cancer cells are at approximately the same rates [2], resulting in a relatively static tumor size. On the other hand, cellular dormancy is resultant of the cancer cells entering a quiescent state of the cell cycle, they neither proliferate nor die [2]. These cells retain the ability to proliferate and later cause tumor recurrence. Cancer cells in cellular dormancy are difficult to treat, for they are resistant to chemotherapy or targeted therapy [3,4]. It is, therefore, essential to understand the biology of cellular dormancy to devise different treatment schemes to cure cancer.

Advanced prostate cancer (PCa) patients are often treated with androgen-deprivation therapy (ADT), which is often very effective since PCas are largely dependent on androgen signaling [5]. However, 10–20% of these patients do experience cancer relapse with the development of castration-resistant prostate cancer (CRPC) [6] within 5 years of follow-up. The long latency period between ADT and CRPC development is characteristic of cancer dormancy. The study of this well-documented clinical phenomenon has been significantly hampered due to the lack of suitable clinical samples and appropriate model systems. To counter this problem, our group was the first to develop and report clinically relevant patient-derived xenograft (PDX) models that developed CRPC after host castration with subsequent latency periods [7]. Recently, we have reported the development of a panel of PDXs from clinical PCa tissues that respond to castration and enter either tumor mass or cellular dormancy by 12 weeks post-castration [8].

Due to the peculiar characteristics of cellular dormancy [2], it is difficult to eliminate these cells with current effective treatments. We hypothesize that these dormant cells should have the ability to overcome immune surveillance for survival. Therefore, learning how they may manipulate immune responses will allow us to devise immunomodulation methods to eliminate these dormant PCa cancer cells, leading to a cure. We analyzed five unique pairs of active and castration-induced dormant PDX samples in this study. RNA sequencing was performed with these samples, and the expression levels of immunomodulatory genes were examined. Using the expression levels of the immune-related genes and the changes of these genes in the dormant state, we reconstructed the intrinsic immunomodulatory ability of the dormant PCa tissues. Our results suggest that these immune evasive mechanisms can be active or passive. Moreover, there are immunomodulatory mechanisms that can be enhanced in the dormant state of PCa. Furthermore, we have identified immune evasion mechanisms that can be utilized by both active and dormant PCa.

## 2. Materials and Methods

### 2.1. Animal Studies

Male severe combined immunodeficient (SCID) mice that were beyond 6 weeks of age were used in these studies. All work performed with the animals was approved by the Animal Care Committee at the University of British Columbia (A12-0024). The patients provided consent for the PCa tumor samples to be developed as PDX. Some of these transplantable PDX lines were reported before [7].

After the PDX samples were grafted under the renal capsules of male SCID mice supplemented with testosterone pellets, as described before [7,8], the xenografts were allowed to grow, and the growth of the PDXs were determined by palpation. When the tumors reached about 500–800 mm^3^ in size, the mice were randomized into 2 groups. One group of mice was euthanized with the tumors harvested as actively growing PCa PDX samples (pre-castration samples). The other group of mice was castrated in addition to the removal of testosterone pellets, the growth (or lack of) of these tumors was monitored with palpation and serum PSA. These mice were then euthanized 12 weeks after the castration and androgen pellet removal, and the remaining tumors were harvested. The selection of this time period was informed by prior characterization of these models [8]. The tumor samples were dissected into sections for snap freezing, processing with RNAlater, or fixation with paraformaldehyde.

### 2.2. Histological and Immunohistochemical Staining

Processing and staining of PDX sections were performed as described before [8]. Briefly, PDX samples were harvested and fixed with 10% formalin for 24 h, after which the samples were processed for paraffin embedding. Sections of 5 μm were cut with a microtome and mounted on glass slides. After de-waxing and rehydration, some slides were stained with hematoxylin and eosin for light microscopy examination. For immunohistochemical staining, the rehydrated sections were submerged in citrate-based unmasking solution (Vector Laboratories, Burlingame, CA, USA) and incubated in boiling water for antigen retrieval. Endogenous peroxidase was blocked with incubation of 3% H_2_O_2_. After washing with PBS, the sections were incubated with Super Block^TM^ blocking buffer before the application of primary antibodies (Ki-67, 1:200, Dako, Santa Clara, CA, USA; anti-cleaved caspase 3, 1:50, Cell Signaling, Danvers, MA, USA) and incubation at 4 ^o^C overnight. After washing with PBS-Tween (PBST), corresponding secondary antibodies conjugated with biotin were applied with subsequent PBST wash. ABC reagent was then applied (Vector Laboratories, Burlingame, CA, USA). Immunoreactivity was visualized with incubation of 3,3′-diaminobenzidine tetra-hydrochloride (DAB) in PBS and 0.01% H_2_O_2_, followed by washing with running water. Hematoxylin was applied as counterstain. The sections were dehydrated after washing and mounted.

### 2.3. Bioinformatic Analyses

Since PDX tissues contain both human cancer cells and mouse stromal cells, we separated human-specific and mouse-specific reads and used the former to estimate gene expressions in cancer cells.

We used genome sequences and gene annotations from both human and mouse (Ensembl GRCh38.90 and GRCm38.90) to build the reference database, and map RNA-Seq reads to this combined reference by STAR 2.6.0a. We used reads only mapped to human genome or transcriptome in the downstream analysis. For all human genes, their read counts were calculated by htseq-count 0.11.2, and their abundances measured by Transcripts Per Millions (TPM) were estimated using kallisto 0.44.0. We also ran DESeq2 1.16.1 to normalize the read counts and obtained fold changes in gene expressions between active and dormant samples in each model. All heatmaps in this article were generated based on normalized read counts using ward.D2 (Ward’s minimum variance method) as the hierarchical clustering method and the Canberra metric.

Immune-related genes in 20 gene groups were selected for the known immunomodulatory functions. The genes were extracted from the HUGO gene nomenclature committee [9] and from the literature [10,11,12,13,14].

### 2.4. Transcriptome Profiling of PCa PDX Samples

To better remove sequencing noises and artifacts from our experiments, we apply the following conditions to remove low-expression genes. For each PDX model, a gene is considered expressed when TPM ≥ 1 and read counts ≥ 10 in either pre-castration or post-castration stage. In this study, we only focused on the genes expressed in at least 3 models. After the removal of low-expression genes, 12,348 protein-coding genes remained in our analysis. Similarly, we considered a gene differentially expressed when it showed a 2-fold change or more toward the same direction (upregulated or downregulated in dormancy) in at least 3 models. We also defined dormant-persistent immune-related genes as those genes with average read counts of 1024 ($log_2^10$) or more in both pre-castration and post-castration stages.

## 3. Results

### 3.1. PDX Models

Five established PDX models were selected for this study due to their ability to enter cellular dormancy upon host castration as a form of ADT [8]. When grafted under the renal capsule of male mouse hosts supplemented with subcutaneous testosterone pellet implants, all established PDXs were able to grow. Upon host castration and removal of testosterone pellets, all PDXs responded with cessation of growth and decrease in tumor volume, as reported before [8]. The PDXs entered a prolonged phase of dormancy, and the viability of the dormant PDX was evident with histological evaluation (below). Molecular characteristics of these PDX models are shown in Table 1. LTL313B and LTL313H were developed from needle biopsies samples collected at different PCa foci [7]. The other models were derived from different individual patients. All PDXs are typical prostatic adenocarcinoma expressing androgen receptor (AR) and PSA. None of the PDX samples expressed neuroendocrine markers, e.g., synaptophysin (SYP) or chromogranin A, indicative of the absence of de novo neuroendocrine cells. Fusion of TMPRSS2 and ERG is commonly detected in PCa [15], and this fusion was present in three out of the five PDX models in this study. PTEN deficiency, another common occurrence in PCa [16], was observed in four out of the five PDX models in this study. The PTEN deficiency in these PDX models was due to heterozygous or homozygous deletion of this gene (Table 1).

### 3.2. Dormancy of Post-Castration PCa PDX Samples

To ensure that the 12-week post-castration PDX samples entered the state of cellular dormancy, we performed histological and immunohistochemical evaluations of the corresponding samples with light microscopy. 

**Figure 1:**
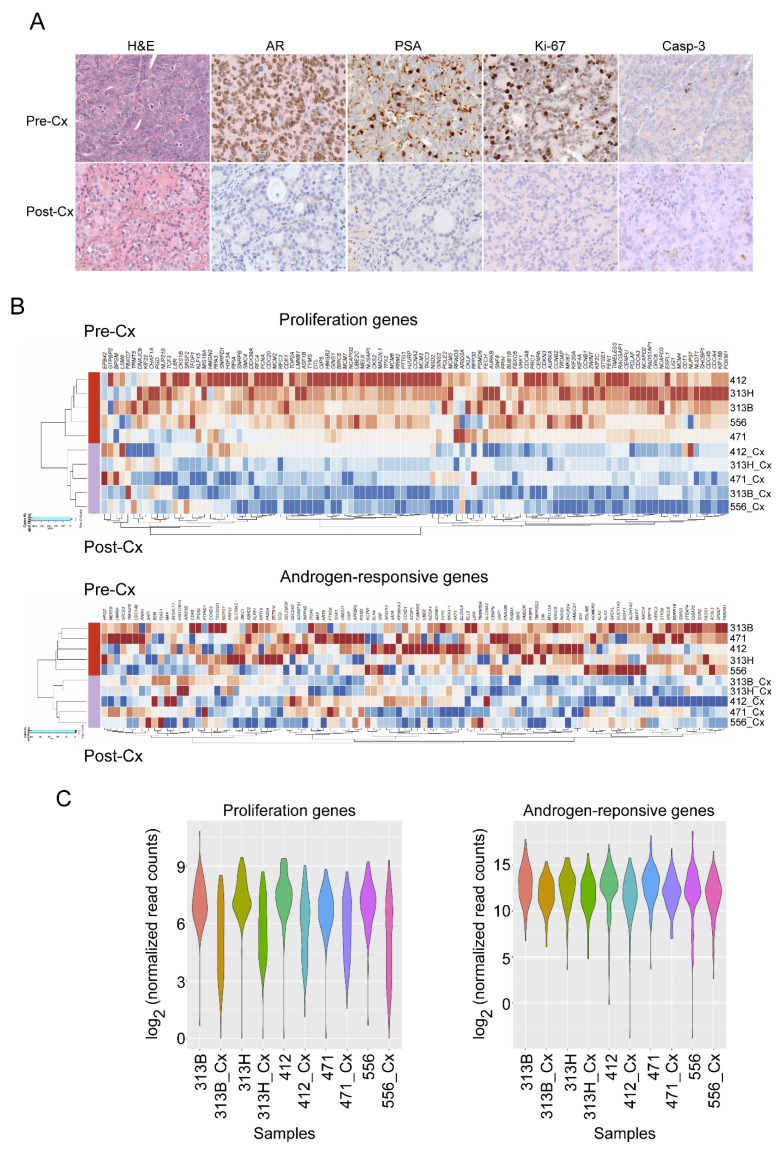
Cellular dormancy of 12-week post-castration samples was confirmed with histology and gene expression analyses. (**A**) Examples of H&E and immunohistochemical staining of pre-castration (Pre-Cx) and 12-week post-castration (Post-Cx) PCa samples (H&E: hematoxylin and eosin; AR: androgen receptor; PSA: prostate-specific antigen; Casp-3: cleaved caspase 3); (**B**) heatmap and unsupervised clustering of proliferation genes and androgen-responsive genes of Pre-Cx and Post-Cx PCa samples; (**C**) violin plots of proliferation genes and androgen-responsive genes of each paired sample. Cx labels indicate the post-castration/dormant samples.

Comparing to the corresponding pre-castration samples, the 12-week post-castration samples displayed significant morphological changes, such as pyknosis (small, condensed nuclei), cytoplasmic vacuolation, and decrease in glandular structure density. Both the mitotic rate and number of Ki-67^+^ decreased significantly (Figure 1A). These observations indicate that most of the human PCa cells were not in the proliferative phase of the cell cycle. At the same time, the 12-week post-castration samples also showed no or a very low-level staining with cleaved caspase 3 (Figure 1A), suggesting that the cells in the post-castration samples were not in apoptosis. Serum PSA was undetectable in the mouse hosts carrying the dormant PDXs. The histological and IHC evaluations were consistent with our expectations that the samples were in the cellular dormant state, as reported before [8].

In addition, we performed RNA sequencing analysis with the 131-proliferation gene set [17] to compare the levels of proliferation between the active and dormant samples. After the removal of low-expression genes, 107 genes remained for the analysis (Supplementary Table S1). The heatmap of the proliferation genes indicated that the 12-week post-castration samples had significantly reduced gene expression levels when compared to the active samples (Figure 1B). Pairwise comparison of the samples with violin plots clearly demonstrated the significant reduction in the expression of the proliferation genes in the dormant samples (Figure 1C). With castration of the PDX-bearing mice, androgen receptor (AR) signaling was expected to be significantly downregulated. We also compared the expression of the androgen-responsive genes (Supplementary Table S2) [18] in the actively growing/pre-castration PDX samples with the PDX samples harvested 12 weeks post-castration. Similar to the expression of the proliferation genes, the androgen-responsive genes were mostly downregulated in the 12-week post-castration samples. This is evident in the unsupervised clustering pattern of the pre-castration and the 12-week post-castration samples (Figure 1B). The pairwise comparison of the androgen-responsive genes with the violin plots also supported the downregulation of these genes in the post-castration samples (Figure 1C), consistent with the downregulation of AR signaling. The results of these analyses served as internal controls of the samples that were used in the RNA sequencing analyses, and the results of these 12-week post-castration samples were consistent with the features of castration-induced cellular dormancy.

### 3.3. A Unique Gene Set That Differentiated Active from Dormant PCa Samples

A set of 363 immune-related genes were selected for the analysis or description of the intrinsic immune landscape of the PDX models. These genes were selected as families of genes with known immunomodulatory functions. There were 20 gene groups selected, including chemokines and their receptors, interferons and their receptors, interleukin and their receptors, tumor necrosis factor superfamily and their receptors, B7 family members, bone morphogenetic factors and their receptors, major histocompatibility complexes and related proteins, regulators of the complement system, mucins, colony-stimulating factors, toll-like receptors, and the genes that are involved in prostaglandin E synthesis. The complete list of selected immune-related genes is presented in Supplementary Table S3. With our workflow, 97 of the immune-related genes passed the thresholding and were subjected to subsequent analyses.

**Figure 2:**
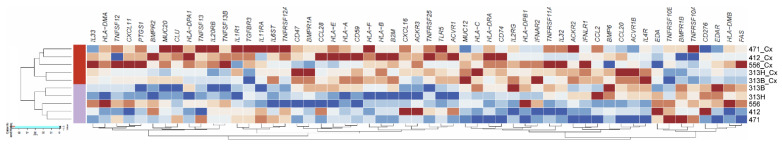
A 54-gene set of immune-related genes that differentiate the active and dormant samples. Cx labels indicate post-castration/dormant samples.

We hypothesized that the tumor microenvironment of the active PCa tumors was significantly different from that of the dormant PCa. Therefore, there might be a difference in the intrinsic immune landscape between the active and dormant samples. To describe such a difference, we constructed a gene set by selecting the most discriminative features between pre-castration and post-castration PDX models using feature importance of random forests classifiers from scikit-learn 0.23.2 [19]. The result was a gene set of 54 genes (Figure 2, Supplementary Table S4). In this gene set were the genes from the major histocompatibility gene group, B7 family members, chemokines, chemokine receptors, interleukins, interleukin receptors, complement system regulators, BMP receptors, TNF receptor superfamily, and PGE_2_ metabolism.

### 3.4. Differentially Expressed Immune-Related Genes

**Figure 3:**
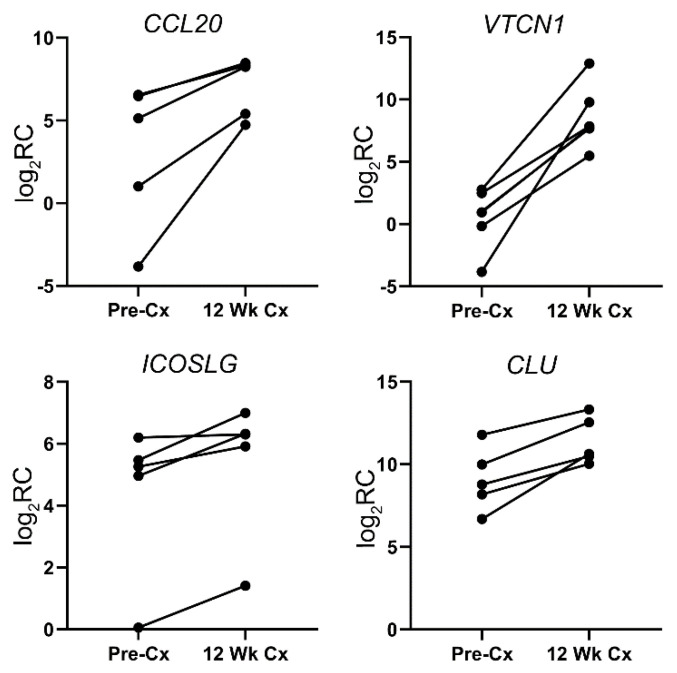
Examples of differentially expressed immune-related genes in dormant PCa samples. Log_2_ values of the read counts (log_2_ RC) of each paired sample (Pre-Cx: pre-castration; 12 Wk Cx: post-castration) with the indicated genes are presented here.

To further highlight the differences in the immune landscape of the dormant PCa tissue from the active ones, we analyzed the differentially expressed immune-related genes. In our analysis, differential expression was defined as >2-fold difference in at least three pairs of the PDX tissues toward the same direction (upregulation or downregulation). With >12,000 human protein-coding genes in our analysis, 776 genes were differentially expressed between the active and dormant states of the PCa PDX tissues. Among the differentially expressed genes, 512 genes were downregulated while 264 genes were upregulated. There were 34 immune-related genes that were differentially expressed, among which 30 genes were upregulated while 4 genes were downregulated. These differentially expressed immune-related genes are listed in Table 2. The differentially expressed genes are from 13 gene groups, while the MHC-related genes were highly represented among the differentially expressed genes. Examples of the differentially expressed genes are presented in Figure 3. *VTCN1*, also known as B7-H4, displays the highest magnitude of differential expression, and the differential expression was present in all five pairs of the PDX tissues.

### 3.5. Dormancy-Persistent Immune-Related Genes

**Figure 4:**
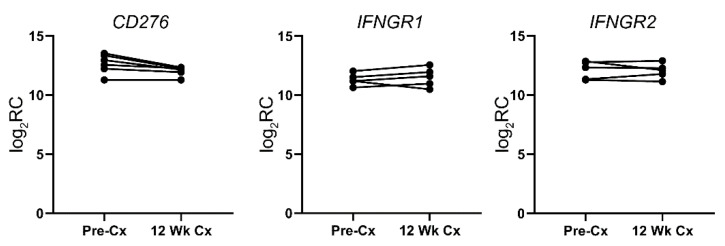
Examples of dormancy-persistent genes in active (Pre-Cx: pre-castration) and dormant (12 Wk Cx: post-castration) PCa samples. Log_2_ values of the read counts (log_2_RC) of the indicated genes are presented here.

Although it is expected that the dormant PCa tissues would employ different immune evasion mechanisms due to the change in the tumor microenvironment, it is conceivable that some of the effective immunomodulatory mechanisms can be utilized by both the active and dormant PCa tissues. To decipher these dormancy-persistent immune evasion mechanisms, we examined immune-related genes that are highly expressed in both active and dormant states of the PCa. Twenty-four immune-related genes were found to be persistently expressed at high levels in the dormant state (Table 3). Within the set of dormancy-persistent immune-related genes, the majority of the genes are surface receptors, including the receptors for interleukins, interferons, tumor necrosis factors, and bone morphogenetic proteins. There are also members of the B7 family, complement regulators, and MHC-related genes. Examples of the dormancy-persistent immune-related genes are presented in Figure 4.

## 4. Discussion

### 4.1. Uniqueness of the Castration-Induced PCa Dormancy PDX Models

Androgen deprivation therapy (ADT) is an effective treatment for advanced PCa. The majority of patients initially respond well to the therapy. The significant reduction in AR signaling often leads to massive PCa cell death. However, most of these patients do experience relapses, and the disease progresses to castration-resistant PCa. Sub-populations of PCa cells must have survived through the drastic reduction in AR signaling and evasion of immune surveillance. However, the phenomenon is particularly difficult to study due to the logistics problem of obtaining the pre- and post-ADT samples from PCa patients. At the same time, available PCa cell lines can only offer a very limited view of the event. Therefore, the study of ADT-induced PCa dormancy has been significantly hampered by the lack of appropriate study models.

The established PDX models utilized in this study have been passaged for generations between the mouse hosts; therefore, it is expected that the non-cancerous human stromal cells were absent in the samples. This included the immune cells. Although the human immune cells are absent, we hypothesized that the PDX PCa cells might express hardwired immunomodulatory molecules that could exert effects on the immune system, whether the immune cells were present or not. Such intrinsic properties of cancer cells have been demonstrated with specific genetic alterations [20]. For instance, it has been demonstrated that PTEN deficiency leads to a significant increase in leukocyte infiltrates due to an upregulation of cytokine profile [21]. We contend that our PDX models provide reductionist models to study the PCa-specific response to ADT and provide a simplified picture of how the PCa cells may modulate the host immunity for their survival.

In our laboratory, we were able to develop a panel of unique PDX models from clinical PCa tissues that are responsive to host castration [7,8]. In particular, we were the first to report PDX cases that went through ADT-induced dormancy and subsequent development into CRPC [7]. Significantly, the patient, from which the sample was obtained, treated with ADT also developed CRPC subsequently. This observation highlights how our PDX models closely resemble the clinical scenarios. Therefore, we believe that we can gain invaluable insights into dormancy biology through studying these unparalleled models. In this study, we utilized five of these PDX models, and they all responded to ADT through surgical castration of the mouse hosts, although they had diverse molecular features, such as different PTEN or TMPRSS2-ERG fusion statuses.

### 4.2. Reconstruction of Intrinsic Immunosuppressive Landscape of Dormant PCa

**Figure 5:**
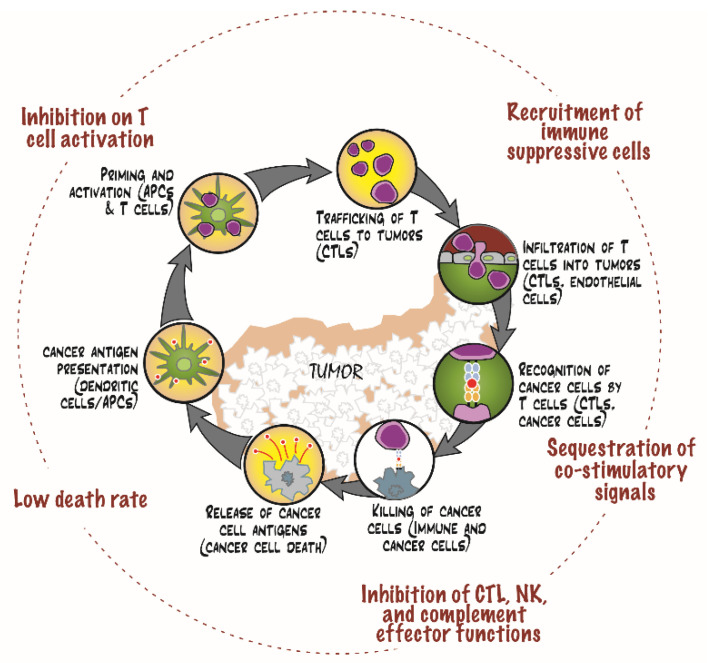
Potential immunosuppressive or immune evasion mechanisms utilized by dormant PCa. Using the proposed cancer-immunity cycle as a framework (inner circle, modified from [22]), the potential immune evasion mechanisms of dormant PCa cells are presented (outer circle, in red). APCs, CTLs, and NK denote antigen-presenting cells, cytotoxic T lymphocytes, and natural killer cells, respectively.

It is well-documented that secreted and cell-surface molecules expressed by cancer cells can and do exert immunomodulatory functions [20]. Although these PDX models do not have human immune cells, studying these secreted and cell-surface molecules expressed by the cells of the PDX models can help reconstruct the potential immune evasion mechanisms utilized by the active and dormant PCa cells. To discuss our discovery in such context, we utilized the cancer-immunity cycle proposed by Chen and Mellman [22], as follows (Figure 5):

#### 4.2.1. Prevention of Effective T Cell Activation

During cellular dormancy, death of PCa cells is a rare event when compared to the active or proliferative state of PCa. Therefore, the rate of release of cancer neoantigen will be much lower. In addition, the low rate of PCa cell death will also limit the amount of alarmins released [23]. Therefore, the dormancy state of PCa is presumably a less immunogenic state of PCa.

One unexpected result with the dormant PCa expression data was the increased expressions of major histocompatibility complexes (classes I and II) and related molecules. During tumor progression, cancer cells expressing MHC molecules are usually selected out through immunoediting [24]. However, it has also been proposed that MHC molecules can modulate tolerance. It was first proposed that tolerated pig liver graft released a tolerogenic form of the MHC molecules [25]. Subsequent work revealed that MHC molecules can be secreted in soluble form, and these molecules are detected in normal and diseased serum or plasma samples [26,27,28]. It is well-documented that proper activation of naïve T cells requires the engagement of the T cell receptor and the antigenic peptide:MHC molecule complex together with costimulatory signals, lest the T cells will be brought into an unresponsive state, known as anergy [29,30,31]. It has been demonstrated that soluble peptide:MHC II molecules can induce T cell anergy in vitro and in vivo [32,33,34]. The presence of the soluble MHC molecules can, therefore, skew the TCR signaling without sufficient co-activation signals, resulting in the induction of anergic T cells during the dormancy state, leading to tolerance toward cancer antigens.

The upregulation of MHC genes is certainly counter-intuitive. It has been demonstrated in PCa cells that the expression levels of IL33 is associated with the expression levels of MHC-I molecules. In addition, there is in vitro evidence that knockdown of IL33 decreases MHC-I expression [35]. Furthermore, IL33 can induce MHC II expression in bone marrow-derived mast cells [36]. These observations are consistent with our findings that IL33 and MHC molecule expressions were increased together in the dormant PCa samples. In addition, IL32, which was upregulated in the dormant PDX samples, can induce monocyte differentiation to DC, leading to upregulation of MHC-I and MHC-II molecules [37]. Whether the effect of IL32 on cancer cells can lead to upregulation of these MHC molecules are yet to be demonstrated. It is known that the expressions of NLRC5 and CIITA are highly associated with the expressions of MHC-I and MHC-II, respectively [38]. Interestingly, these molecules are generally downregulated in tumors, yet they were upregulated in the dormant PDX samples (Supplementary Figure S1). The upregulation of these genes was consistent with our observations that both MHC-I and MHC-II molecules were upregulated. When we examined the protein expression level of HLA-A, an MHC-I molecule, with IHC in the actively growing PCa PDX and dormant PCa PDX, HLA-A was found to be upregulated in the dormant PDX samples (Supplementary Figure S2).

#### 4.2.2. Recruitment of Immunosuppressive Cells to Build an Immune Privileged Niche

Among the over-expressed genes, there are gene products that can recruit and modulate immune cell functions that create an immunosuppressive microenvironment. For instance, CCL20, upregulated in the dormant PDX samples, can recruit CCR6^+^ Treg cells to the microenvironment [39]. Another cytokine CXCL11 is also upregulated in the dormant samples. Although its role in tumor biology is unclear at the moment, this cytokine has been shown to drive IL-10^hi^ Treg polarization [40], which may inhibit anticancer immunity. In addition, IL-33 has been shown to recruit and induce pro-tumorigenic tumor-associated macrophages [41,42]. Together, these observations suggest that through cytokine production, the dormant PCa cells can recruit immunosuppressive cells to create a niche for the evasion of immune surveillance.

#### 4.2.3. Inhibition of the Immune Effector Functions

In addition to the potential of inhibiting T cell activation, MHC molecules have also been shown to modulate the effector functions of immune cells. Soluble MHC I molecules are able to induce apoptotic death of activated alloreactive CD8^+^ cytotoxic T cells and NK cells [43,44,45]. The dormant PCa may employ this immune evasion mechanism, therefore, to eliminate CD8^+^ CTLs that are reactive to PCa antigens. In addition, the increased expression of non-classical MHC-I molecules HLA-E and HLA-F in the dormant PDX can enable the engagement of inhibitory receptors on NK and activated T cells [46,47,48,49], and thereby inhibiting their cytotoxic effector functions [47,50,51,52]. It is interesting to note that senescent cells, which share similarities with dormant cells [3], can utilize HLA-E to evade NK and T cell cytotoxicity [53], while HLA-F have been postulated to regulate fetomaternal immune homeostasis [54]. Perhaps the dormant PCa cells are hijacking these physiological mechanisms to evade immune surveillance. There is also recent evidence that the expression of MHC-II molecules can suppress effector functions of NK and T cells through interactions with LAG3 and/or FCRL6 on these immune cells [55,56,57,58,59].

There has been immense interest in understanding and exploiting immune checkpoint mechanisms due to recent successes of various immune checkpoint inhibitors [10]. We have examined the expression levels of the B7 family members, among which many are checkpoint molecules [11,60]. Current evidence suggests that B7 family members can both positively and negatively regulate immunity [61]. PD-L1 has been approved by FDA as a biomarker for immune checkpoint inhibitors that block the PD-1/PD-L1 axis for a variety of cancers [62]. However, PCa is not one of the approved indications. PD-L2 has recently been suggested as a therapeutic target for PCa [63]. Interestingly, however, the TPM values of *CD274* (PD-L1) and *PDCD1LG2 (*PD-L2) were very low, suggesting no or low expressions of these B7 family members. On the other hand, we found that the expression levels of *B7-H3* were consistently high in all the active and dormant PCa samples. B7-H3 can exert its inhibitory influence on Th1 response [64], T cell effector function [65], and NK cytolytic activity [66], potentially promoting immune evasion. Consistent with its potential role in immune evasion and, thereby, promoting tumor progression, B7-H3 expression has been shown to correlate with tumor aggressiveness [67,68] and poor clinical outcomes [69,70] in various cancers. We have also detected increased expression of *VTCN1*, which encodes B7-H4, in all paired dormant PCa samples. B7-H4 has been shown to negatively regulate T cell immunity [71,72,73]. Blockade of B7-H4 in animal models has been shown to enhance anticancer immune responses [74,75], demonstrating the in vivo immunosuppressive function of B7-H4. In PCa, high immunoreactivity against B7-H4 in clinical FFPE tissues is associated with increased risk of recurrence [70]. Therefore, with increased expression of B7-H4 in all five PDX models during dormancy, it is plausible that B7-H4 may play a role in mediating immune evasion during dormancy. It is interesting to note that, in an animal model, B7-H4 expression on antigen-presenting cells in the tumors was responsible for inhibiting cytotoxic function of CD8^+^ cells and promoting T cell exhaustion [76]. With the unexpected increase in MHC II molecules in the dormant PCa cells together with the increased expression of B7-H4, one might wonder if the dormant PCa cells can “pretend” to be APCs in the aforementioned context to promote T cell exhaustion.

Prostaglandin E_2_ (PGE_2_) is a lipid that has been demonstrated to possess immunomodulatory activities at many different levels [77]. The net production of PGE_2_ is generally regulated by the synthetic activities of cyclooxygenases (encoded by *PTGS1* and *PTGS2*) and degradation by 15-hydroxyprostaglandin dehydrogenase (encoded by *HPGD*) [78]. In our analysis, increased expression of cyclooxygenase 1 and prostaglandin E synthase together with the decreased expression of 15-hydroxyprostaglandin dehydrogenase was observed, suggesting a net increase in PGE_2_. It is, therefore, conceivable that dormant PCa cells may utilize the immunosuppressive effects of PGE_2_ to evade anticancer immunity, including suppression of NK and CTL cytolytic activities [79,80,81] and promotion of Treg and MDSC activities [82,83,84,85].

#### 4.2.4. Sequestration of Activating Cytokines from Immune Cells

It is intriguing that the dormant PCa cells are expressing many receptors that are usually expressed on immune cells for costimulatory function, such as IL-1 receptor and IFNγ receptor. We hypothesize that these receptors are expressed to sequester the stimulatory ligands, be they soluble or membrane-bound, from the immune cells. Consequently, the immune cells will be deprived of essential activation signals for their effector functions. The sequestration of such stimulatory signal may promote T cell exhaustion [86]. An example is IFNγ receptor 1 (*IFNGR1*), a subunit of the IFNγ receptor that was highly expressed in the dormant PCa samples. IFNγ is an essential regulator of antitumor immunity whose functions include the promotion of CTL differentiation and function [87,88], inhibition of pro-tumorigenic M2 macrophage polarization [89], and differentiation and function of Treg cells [90,91]. Therefore, the sequestration of IFNγ will understandably hamper antitumor immunity. To our knowledge, sequestration of pro-antitumor immunity as an immune evasion mechanism has not been explored in any details and further study is warranted.

#### 4.2.5. Inhibition of Complement-Mediated Injury

According to our analysis, *CD46* and *CD59* were consistently expressed at high levels whether the PCa were in the active or dormant state. These surface proteins are well-documented for their ability to inhibit the activation of the complement system and the activity of the complement membrane complex, respectively [92]. In addition, the expression of clusterin, a secreted protein that inhibits the formation of the complement membrane attack complex [93], was increased in dormant PCa tissues. Together, the dormant PCa tissues seem to be able to suppress anticancer functions of the complement system [94] at multiple levels.

These observations suggest that dormant PCa cells possess the potential to evade immunosurveillance in both active and passive manners. The dormant PCa cells may actively fashion the tumor microenvironment through recruitment and induction of suppressive immune cells, thereby creating an immunosuppressive niche. On the other hand, the dormant PCa cells may also passively avoid the potential harm exerted from the antitumor immunity, such as inhibition of cytotoxicity from CTL and NK cells.

### 4.3. Current Biomarkers for Immunotherapy

Currently, PD-L1 serves as a predictive biomarker for immune checkpoint inhibitors that block the PD-1/PD-L1 axis, such as pembrolizumab and nivolumab [62]. There have been many different tumor types, including lung cancer, melanoma, bladder cancers, and renal cancers, approved by FDA for these immune checkpoint inhibitors. We have examined the expression levels of all B7 family members, including PD-L1, in our active and dormant PDX samples. The TPM values of *CD274* (PD-L1) and *PDCD1LG2* (PD-L2), both of which are ligands of PD-1, were very low. According to our data, blocking the PD-1/PD-L1 or PD-L2 axis may not be a plausible attempt to re-activate the immune cells for the elimination of dormant cells. Intriguingly, *VCTN1*, which encodes B7-H4, a known immunosuppressor in the B7 family, was found to be upregulated in all the dormant PCa PDX when compared to their corresponding active PDX samples, while the expression levels of B7-H3 were consistently high in the active and dormant PCa PDX samples. Whether these B7 family members can be targeted for the elimination of dormant PCa cells remains to be validated.

The mismatch repair (MMR) pathway is essential for DNA repair and maintaining genome stability. Deficiency in one or more of the four key MMR genes, *MLH1*, *PMS2*, *MSH2*, and *MSH6*, results in microsatellite instability (MSI-H) and is associated with high mutational load [95]. MMR deficiency was found to be a good predictor of response to the immune checkpoint inhibitor pembrolizumab [96]. Therefore, pembrolizumab was approved by FDA to treat unresectable or metastatic MMR-deficient solid tumors in 2017. MMR-deficiency should be associated with higher tumor mutational burden (TMB) [97]. Tumors with high TMB (TBM-H), defined as >10 mutations/Mb, are thought to correlate with higher probability of generating neoantigens and are therefore more immunogenic. Recently, TMB-H has been approved by FDA as an indication for anti-PD-1 pembrolizumab based on the results of KEYNOTE-158. However, further study suggests that TMB-H may only be predictive of the immune checkpoint inhibitor response in a limited number of cancers, such as lung cancers, bladder cancers, and melanoma [98]. Although TMB-H PCa is associated with lower overall survival [99], TMB of PCa does not correlate with the response to immune checkpoint inhibitors [98]. There is still much ongoing effort to determine the applicability of these biomarkers across various tumor types, whether these findings are applicable to ADT-induced dormancy remains to be seen. In our study, we have identified a panel of soluble immunomodulatory factors, such as chemokines and cytokines, that may contribute to the survival of dormant PCa from immunosurveillance. These soluble factors, if validated, may not simply be exploited for novel immunotherapy strategies, but they may also serve as biomarkers for these novel immunotherapy strategies.

### 4.4. Limitation of the Current Work and Future Directions

Our current work provides a descriptive landscape of the intrinsic immunomodulatory potential of castration-induced dormant PCa. We acknowledge two major areas of limitation of our current work, and these limitations should prompt future endeavors to further investigate the immunologic landscape of dormant PCa and the potential to exploit these properties of dormant PCa for the elimination of these cells. One, since immunocompromised mice are required for the development of PDX, the absence of functional immune cells renders the validation of the immunomodulatory potential identified in this study impossible. To validate or further study these immunomodulatory potentials, it will be best to utilize autologous humanized PDX models, similar to what was described by Gitto et al. [100]. Second, the persistently expressed or upregulated immune-related genes in the dormant PDX samples will need to be validated with clinical specimens. Access to such specimens is a challenge since biopsy is not generally required after ADT. This obstacle may be overcome through accessing samples with prospective clinical studies that require such specimens. Alternatively, the effect of the soluble factors, such as the interleukins and cytokines, can first be examined since consent may be easier to obtain for non-invasive procedures. The study of these soluble immune-related molecules may also have the potential to serve as biomarkers or therapeutic targets.

## 5. Conclusions

In conclusion, we have employed five rare and exceedingly valuable hormone-responsive patient-derived xenografts to study the intrinsic immune landscapes of the active and castration-induced dormant PCa. Our results indicate that dormant PCa may utilize a host of soluble and cell surface molecules to exert immunomodulatory effects. With the reconstruction of the potential immune evasion mechanisms that can be utilized by the dormant PCa cells, this work provides a framework for further studies. The insights provided by this study also implicate that the recruitment of the immune system to eliminate the dormant PCa cells will likely require a combination approach.

## Figures and Tables

**Table 1:** Molecular characteristics of the PDX models.

LTL ID	AR	PSA	SYP	ERG	TMPRSS2-ERG Fusion	PTEN	*PTEN* Status
LTL313B	+	+	−	+	+	−	−/−
LTL313H	+	+	−	+	+	−	−/−
LTL412	+	+	−	−	−	−	+/−
LTL471	+	+	−	−	−	+	+/+
LTL556	+	+	−	+	+	−	−/−

**Table 2:** Differentially expressed genes in dormant PDX samples.

Family Name	Gene	Full Name	Differential Expression
BMP Receptors	*BMPR1B*	Bone Morphogenetic Protein Receptor Type 1B	Down
Chemokine	*CCL20*	Chemokine (C-C motif) ligand 20	Up
	*CXCL11*	C-X-C motif chemokine 11	Up
Complement	*CLU*	Clusterin	Up
Colony Stimulating Factor	*CSF1*	Colony Stimulating Factor 1	Up
MHC-related	*HLA-A*	MHC I, A	UP
	*HLA-B*	MHC I, B	Up
	*HLA-E*	non-classical MHC I, alpha chain E	Up
	*HLA-F*	non-classical MHC I, alpha chain F	Up
	*CD7*4	Invariant chain	Up
	*B2M*	β2 microglobulin	Up
	*HLA-DPA1*	MHC II, DP alpha chain	Up
	*HLA-DPB1*	MHC II, DP Beta 1	Up
	*HLA-DRA*	MHC II, DR alpha chain	Up
	*HLA-DMA*	MHC II, DM alpha chain	Up
	*HLA-DMB*		Down
Interleukin	*IL32*	Interleukin 32	Up
	*IL33*	Interleukin 33	Up
Interleukin receptors	*IL6R*	Interleukin 6 Receptor	Up
	*IL5RA*	Interleukin 5 Receptor Subunit Alpha	Up
	*IL2RG*	Interleukin 2 Receptor Subunit Gamma	Up
B7 family	*VTCN1*	V-Set Domain Containing T Cell Activation Inhibitor 1	Up
	*ICOSLG*	Inducible T Cell Costimulator Ligand	Up
	*NCR3LG1*	Natural Killer Cell Cytotoxicity Receptor 3 Ligand 1	Up
Mucin	*MUC12*	Mucin 12, Cell Surface Associated	Up
TGFBR	*TGFBR3*	Transforming Growth Factor Beta Receptor 3	Up
TLR	*TLR5*	Toll-like receptor 5	Up
TNFRSF	*TNFRSF25*	TNF Receptor Superfamily Member 25	Up
	*TNFRSF12A*	TNF Receptor Superfamily Member 12A	Up
	*TNFRSF11B*	TNF receptor superfamily member 11B	Up
PGE_2_ metabolism	*PTGS2*	Prostaglandin-Endoperoxide Synthase 2	Down
	*PTGS1*	Prostaglandin-Endoperoxide Synthase 1	Up
	*PTGES*	Prostaglandin E Synthase	Up
	*HPGD*	15-Hydroxyprostaglandin Dehydrogenase	Down

**Table 3:** Abundantly expressed immune-related genes in both active and dormant PDX samples.

Family Name	Gene	Full Name
BMP Receptors	*BMPR1A*	Bone morphogenetic protein receptor type 1A
	*BMPR2*	Bone morphogenetic protein receptor type 2
	*ACVR1B*	Activin A receptor type 1B
Complement regulator	*CD46*	CD46 molecule
	*CD59*	CD59 molecule
MHC-related	*HLA-C*	Major histocompatibility complex, class I, C
IL Receptors	*IL13RA1*	Interleukin 13 receptor subunit alpha 1
	*IL17RA*	Interleukin 17 receptor A
	*IL17RD*	Interleukin 17 receptor D
	*IL20RA*	Interleukin 20 receptor subunit alpha
	*IL4R*	interleukin 4 receptor
	*IL6ST*	interleukin 6 signal transducer
	*IL17RC*	interleukin 17 receptor C
	*IL1R1*	interleukin 1 receptor type 1
B7 family	*CD276*	B7-H3
IFN Receptor	*IFNGR2*	interferon gamma receptor 2
	*IFNAR1*	interferon alpha and beta receptor subunit 1
	*IFNGR1*	interferon gamma receptor 1
TGFBR	*TGFBR1*	transforming growth factor beta receptor 1
TNFSF	*TNFSF10*	TNF superfamily member 10
TNFRSF	*TNFRSF19*	TNF receptor superfamily member 19
	*LTBR*	lymphotoxin beta receptor
	*TNFRSF14*	TNF receptor superfamily member 14
	*TNFRSF21*	TNF receptor superfamily member 21

## Data Availability

The data presented in this study are available on request from the corresponding author.

## Supplementary Materials

The following supporting information can be downloaded at: https://www.mdpi.com/article/10.3390/cells11091550/s1, Supplementary Figure S1: Normalized expression (log_2_ scale) of *CIITA* and *NLRC5*; Supplementary Figure S2: A. Examples of IHC staining of active (Pre-Cx) and dormant (Post-Cx) PDX tissues; Supplementary Table S1: List of proliferation genes; Supplementary Table S2: List of androgen response genes; Supplementary Table S3: List of Immune-related genes used in the analysis; Supplementary Table S4: List of genes that separated the active and dormant samples; Supplementary Methods.

## References

[1] Aguirre-Ghiso J.A. (2007). Models, mechanisms and clinical evidence for cancer dormancy. Nat. Rev. Cancer.

[2] Endo H., Inoue M. (2019). Dormancy in cancer. Cancer Sci..

[3] Kurppa K.J., Liu Y., To C., Zhang T., Fan M., Vajdi A., Knelson E.H., Xie Y., Lim K., Cejas P. (2020). Treatment-Induced Tumor Dormancy through YAP-Mediated Transcriptional Reprogramming of the Apoptotic Pathway. Cancer Cell.

[4] Phan T.G., Croucher P.I. (2020). The dormant cancer cell life cycle. Nat. Rev. Cancer.

[5] Crawford E.D., Heidenreich A., Lawrentschuk N., Tombal B., Pompeo A.C.L., Mendoza-Valdes A., Miller K., Debruyne F.M.J., Klotz L. (2019). Androgen-targeted therapy in men with prostate cancer: Evolving practice and future considerations. Prostate Cancer Prostatic Dis..

[6] Kirby M., Hirst C., Crawford E.D. (2011). Characterising the castration-resistant prostate cancer population: A systematic review. Int. J. Clin. Pract..

[7] Lin D., Wyatt A.W., Xue H., Wang Y.Y., Dong X., Haegert A., Wu R., Brahmbhatt S., Mo F., Jong L. (2014). High fidelity patient-derived xenografts for accelerating prostate cancer discovery and drug development. Cancer Res..

[8] Dong X., Xue H., Mo F., Lin Y.-Y., Lin D., Wong N.K.Y., Sun Y., Wilkinson S., Ku A.T., Hao J. (2022). Modeling androgen deprivation therapy-induced prostate cancer dormancy and its clinical implications. Mol. Cancer Res..

[9] Braschi B., Denny P., Gray K., Jones T., Seal R., Tweedie S., Yates B., Bruford E. (2019). Genenames.org: The HGNC and VGNC resources in 2019. Nucleic Acids Res..

[10] Topalian S.L., Drake C.G., Pardoll D.M. (2015). Immune checkpoint blockade: A common denominator approach to cancer therapy. Cancer Cell.

[11] Ceeraz S., Nowak E.C., Noelle R.J. (2013). B7 family checkpoint regulators in immune regulation and disease. Trends Immunol..

[12] Kuczma M., Kraj P. (2015). Bone Morphogenetic Protein Signaling Regulates Development and Activation of CD4(+) T Cells. Vitam. Horm..

[13] Bhatia R., Gautam S.K., Cannon A., Thompson C., Hall B.R., Aithal A., Banerjee K., Jain M., Solheim J.C., Kumar S. (2019). Cancer-associated mucins: Role in immune modulation and metastasis. Cancer Metastasis Rev..

[14] Ching M.M., Reader J., Fulton A.M. (2020). Eicosanoids in Cancer: Prostaglandin E(2) Receptor 4 in Cancer Therapeutics and Immunotherapy. Front. Pharmacol..

[15] Tomlins S.A., Laxman B., Varambally S., Cao X., Yu J., Helgeson B.E., Cao Q., Prensner J.R., Rubin M.A., Shah R.B. (2008). Role of the TMPRSS2-ERG gene fusion in prostate cancer. Neoplasia.

[16] Jamaspishvili T., Berman D.M., Ross A.E., Scher H.I., De Marzo A.M., Squire J.A., Lotan T.L. (2018). Clinical implications of PTEN loss in prostate cancer. Nat. Rev. Urol..

[17] Venet D., Dumont J.E., Detours V. (2011). Most random gene expression signatures are significantly associated with breast cancer outcome. PLoS Comput. Biol..

[18] Nelson P.S., Clegg N., Arnold H., Ferguson C., Bonham M., White J., Hood L., Lin B. (2002). The program of androgen-responsive genes in neoplastic prostate epithelium. Proc. Natl. Acad. Sci. USA.

[19] Pedregosa F., Varoquaux G., Gramfort A., Michel V., Thirion B., Grisel O., Blondel M., Prettenhofer P., Weiss R., Dubourg V. (2011). Scikit-learn: Machine Learning in Python. J. Mach. Learn. Res..

[20] Wellenstein M.D., de Visser K.E. (2018). Cancer-Cell-Intrinsic Mechanisms Shaping the Tumor Immune Landscape. Immunity.

[21] Ying H., Elpek K.G., Vinjamoori A., Zimmerman S.M., Chu G.C., Yan H., Fletcher-Sananikone E., Zhang H., Liu Y., Wang W. (2011). PTEN is a major tumor suppressor in pancreatic ductal adenocarcinoma and regulates an NF-κB-cytokine network. Cancer Discov..

[22] Chen D.S., Mellman I. (2013). Oncology meets immunology: The cancer-immunity cycle. Immunity.

[23] Garg A.D., Agostinis P. (2017). Cell death and immunity in cancer: From danger signals to mimicry of pathogen defense responses. Immunol. Rev..

[24] Dunn G.P., Old L.J., Schreiber R.D. (2004). The three Es of cancer immunoediting. Annu. Rev. Immunol..

[25] Calne R.Y., Sells R.A., Pena J.R., Davis D.R., Millard P.R., Herbertson B.M., Binns R.M., Davies D.A.L. (1969). Induction of immunological tolerance by porcine liver allografts. Nature.

[26] Jendro M., Goronzy J.J., Weyand C.M. (1991). Structural and functional characterization of hla-dr molecules circulating in the serum. Autoimmunity.

[27] Haga J.A., She J.X., Kao K.J. (1991). Biochemical characterization of 39-kDa class I histocompatibility antigen in plasma: A secretable membrane protein derived from transmembrane domain deletion. J. Biol. Chem..

[28] Krangel M.S. (1987). Two forms of HLA class I molecules in human plasma. Hum. Immunol..

[29] Jenkins M.K., Pardoll D.M., Mizuguchi J., Quill H., Schwartz R.H. (1987). T-cell unresponsiveness in vivo and in vitro: Fine specificity of induction and molecular characterization of the unresponsive state. Immunol. Rev..

[30] Boussiotis V.A., Gribben J.G., Freeman G.J., Nadler L.M. (1994). Blockade of the CD28 co-stimulatory pathway: A means to induce tolerance. Curr. Opin. Immunol..

[31] Sloan-Lancaster J., Evavold B.D., Allen P.M. (1993). Induction of T-cell anergy by altered T-cell-receptor ligand on live antigen-presenting cells. Nature.

[32] Casares S., Hurtado A., McEvoy R.C., Sarukhan A., von Boehmer H., Brumeanu T.-D. (2002). Down-regulation of diabetogenic CD4+ T cells by a soluble dimeric peptide-MHC class II chimera. Nat. Immunol..

[33] O’Herrin S.M., Slansky J.E., Tang Q., Markiewicz M.A., Gajewski T.F., Pardoll D.M., Schneck J.P., Bluestone J.A. (2001). Antigen-specific blockade of T cells in vivo using dimeric MHC peptide. J. Immunol..

[34] Thomas S., Kumar R., Preda-Pais A., Casares S., Brumeanu T.-D. (2003). A model for antigen-specific T-cell anergy: Displacement of CD4-p56(lck) signalosome from the lipid rafts by a soluble, dimeric peptide-MHC class II chimera. J. Immunol..

[35] Saranchova I., Han J., Huang H., Fenninger F., Choi K.B., Munro L., Pfeifer C., Welch I., Wyatt A.W., Fazli L. (2016). Discovery of a Metastatic Immune Escape Mechanism Initiated by the Loss of Expression of the Tumour Biomarker Interleukin-33. Sci. Rep..

[36] Ito T., Egusa C., Maeda T., Numata T., Nakano N., Nishiyama C., Tsuboi R. (2015). IL-33 promotes MHC class II expression in murine mast cells. Immun. Inflamm. Dis..

[37] Schenk M., Krutzik S.R., Sieling P.A., Lee D.J., Teles R.M.B., Ochoa M.T., Komisopoulou E., Sarno E.N., Rea T.H., Graeber T.G. (2012). NOD2 triggers an interleukin-32-dependent human dendritic cell program in leprosy. Nat. Med..

[38] Nash G., Paidimuddala B., Zhang L. (2022). Structural aspects of the MHC expression control system. Biophys. Chem..

[39] Liu J., Zhang N., Li Q., Zhang W., Ke F., Leng Q., Wang H., Chen J., Wang H. (2011). Tumor-associated macrophages recruit CCR6+ regulatory T cells and promote the development of colorectal cancer via enhancing CCL20 production in mice. PLoS ONE.

[40] Zohar Y., Wildbaum G., Novak R., Salzman A.L., Thelen M., Alon R., Barsheshet Y., Karp C.L., Karin N. (2014). CXCL11-dependent induction of FOXP3-negative regulatory T cells suppresses autoimmune encephalomyelitis. J. Clin. Investig..

[41] Fang M., Li Y., Huang K., Qi S., Zhang J., Zgodzinski W., Majewski M., Wallner G., Gozdz S., Macek P. (2017). IL33 promotes colon cancer cell stemness via JNK activation and macrophage recruitment. Cancer Res..

[42] Wang K., Shan S., Yang Z., Gu X., Wang Y., Wang C., Ren T. (2017). IL-33 blockade suppresses tumor growth of human lung cancer through direct and indirect pathways in a preclinical model. Oncotarget.

[43] Spaggiari G.M., Contini P., Carosio R., Arvigo M., Ghio M., Oddone D., Dondero A., Zocchi M.R., Puppo F., Indiveri F. (2002). Soluble HLA class I molecules induce natural killer cell apoptosis through the engagement of CD8: Evidence for a negative regulation exerted by members of the inhibitory receptor superfamily. Blood.

[44] Takikita M., Altekruse S., Lynch C.F., Goodman M.T., Hernandez B.Y., Green M., Cozen W., Cockburn M., Sibug Saber M., Topor M. (2009). Associations between Selected Biomarkers and Prognosis in a Population-Based Pancreatic Cancer Tissue Microarray. Cancer Res..

[45] Contini P., Ghio M., Poggi A., Filaci G., Indiveri F., Ferrone S., Puppo F. (2003). Soluble HLA-A,-B,-C and -G molecules induce apoptosis in T and NK CD8+ cells and inhibit cytotoxic T cell activity through CD8 ligation. Eur. J. Immunol..

[46] Braud V.M., Allan D.S., O’Callaghan C.A., Söderström K., D’Andrea A., Ogg G.S., Lazetic S., Young N.T., Bell J.I., Phillips J.H. (1998). HLA-E binds to natural killer cell receptors CD94/NKG2A, B and C. Nature.

[47] Lepin E.J., Bastin J.M., Allan D.S., Roncador G., Braud V.M., Mason D.Y., van der Merwe P.A., McMichael A.J., Bell J.I., Powis S.H. (2000). Functional characterization of HLA-F and binding of HLA-F tetramers to ILT2 and ILT4 receptors. Eur. J. Immunol..

[48] Dulberger C.L., McMurtrey C.P., Hölzemer A., Neu K.E., Liu V., Steinbach A.M., Garcia-Beltran W.F., Sulak M., Jabri B., Lynch V.J. (2017). Human Leukocyte Antigen F Presents Peptides and Regulates Immunity through Interactions with NK Cell Receptors. Immunity.

[49] Goodridge J.P., Burian A., Lee N., Geraghty D.E. (2013). HLA-F and MHC class I open conformers are ligands for NK cell Ig-like receptors. J. Immunol..

[50] Le Dréan E., Vély F., Olcese L., Cambiaggi A., Guia S., Krystal G., Gervois N., Moretta A., Jotereau F., Vivier E. (1998). Inhibition of antigen-induced T cell response and antibody-induced NK cell cytotoxicity by NKG2A: Association of NKG2A with SHP-1 and SHP-2 protein-tyrosine phosphatases. Eur. J. Immunol..

[51] Moon J.J., Huang B., Irvine D.J. (2012). Engineering nano- and microparticles to tune immunity. Adv. Mater..

[52] Lin A., Yan W.-H. (2019). The Emerging Roles of Human Leukocyte Antigen-F in Immune Modulation and Viral Infection. Front. Immunol..

[53] Pereira B.I., Devine O.P., Vukmanovic-Stejic M., Chambers E.S., Subramanian P., Patel N., Virasami A., Sebire N.J., Kinsler V., Valdovinos A. (2019). Senescent cells evade immune clearance via HLA-E-mediated NK and CD8(+) T cell inhibition. Nat. Commun..

[54] Persson G., Jørgensen N., Nilsson L.L., Andersen L.H.J., Hviid T.V.F. (2020). A role for both HLA-F and HLA-G in reproduction and during pregnancy?. Hum. Immunol..

[55] Huard B., Prigent P., Tournier M., Bruniquel D., Triebel F. (1995). CD4/major histocompatibility complex class II interaction analyzed with CD4- and lymphocyte activation gene-3 (LAG-3)-Ig fusion proteins. Eur. J. Immunol..

[56] Baixeras E., Huard B., Miossec C., Jitsukawa S., Martin M., Hercend T., Auffray C., Triebel F., Piatier-Tonneau D. (1992). Characterization of the lymphocyte activation gene 3-encoded protein. A new ligand for human leukocyte antigen class II antigens. J. Exp. Med..

[57] Huard B., Tournier M., Hercend T., Triebel F., Faure F. (1994). Lymphocyte-activation gene 3/major histocompatibility complex class II interaction modulates the antigenic response of CD4+ T lymphocytes. Eur. J. Immunol..

[58] Schreeder D.M., Cannon J.P., Wu J., Li R., Shakhmatov M.A., Davis R.S. (2010). Cutting edge: FcR-like 6 is an MHC class II receptor. J. Immunol..

[59] Johnson D.B., Nixon M.J., Wang Y., Wang D.Y., Castellanos E., Estrada M.V., Ericsson-Gonzalez P.I., Cote C.H., Salgado R., Sanchez V. (2018). Tumor-specific MHC-II expression drives a unique pattern of resistance to immunotherapy via LAG-3/FCRL6 engagement. JCI Insight.

[60] Ni L., Dong C. (2017). New B7 Family Checkpoints in Human Cancers. Mol. Cancer Ther..

[61] Janakiram M., Shah U.A., Liu W., Zhao A., Schoenberg M.P., Zang X. (2017). The third group of the B7-CD28 immune checkpoint family: HHLA2, TMIGD2, B7x, and B7-H3. Immunol. Rev..

[62] Davis A.A., Patel V.G. (2019). The role of PD-L1 expression as a predictive biomarker: An analysis of all US Food and Drug Administration (FDA) approvals of immune checkpoint inhibitors. J. Immunother. Cancer.

[63] Zhao S.G., Lehrer J., Chang S.L., Das R., Erho N., Liu Y., Sjöström M., Den R.B., Freedland S.J., Klein E.A. (2019). The Immune Landscape of Prostate Cancer and Nomination of PD-L2 as a Potential Therapeutic Target. J. Natl. Cancer Inst..

[64] Suh W.K., Gajewska B.U., Okada H., Gronski M.A., Bertram E.M., Dawicki W., Duncan G.S., Bukczynski J., Plyte S., Elia A. (2003). The B7 family member B7-H3 preferentially downregulates T helper type 1-mediated immune responses. Nat. Immunol..

[65] Prasad D.V.R., Nguyen T., Li Z., Yang Y., Duong J., Wang Y., Dong C. (2004). Murine B7-H3 is a negative regulator of T cells. J. Immunol..

[66] Castriconi R., Dondero A., Augugliaro R., Cantoni C., Carnemolla B., Sementa A.R., Negri F., Conte R., Corrias M.V., Moretta L. (2004). Identification of 4Ig-B7-H3 as a neuroblastoma-associated molecule that exerts a protective role from an NK cell-mediated lysis. Proc. Natl. Acad. Sci. USA.

[67] Wu S., Zhao X., Wu S., Du R., Zhu Q., Fang H., Zhang X., Zhang C., Zheng W., Yang J. (2016). Overexpression of B7-H3 correlates with aggressive clinicopathological characteristics in non-small cell lung cancer. Oncotarget.

[68] Benzon B., Zhao S.G., Haffner M.C., Takhar M., Erho N., Yousefi K., Hurley P., Bishop J.L., Tosoian J., Ghabili K. (2017). Correlation of B7-H3 with androgen receptor, immune pathways and poor outcome in prostate cancer: An expression-based analysis. Prostate Cancer Prostatic Dis..

[69] Crispen P.L., Sheinin Y., Roth T.J., Lohse C.M., Kuntz S.M., Frigola X., Thompson R.H., Boorjian S.A., Dong H., Leibovich B.C. (2008). Tumor cell and tumor vasculature expression of B7-H3 predict survival in clear cell renal cell carcinoma. Clin. Cancer Res. Off. J. Am. Assoc. Cancer Res..

[70] Zang X., Thompson R.H., Al-Ahmadie H.A., Serio A.M., Reuter V.E., Eastham J.A., Scardino P.T., Sharma P., Allison J.P. (2007). B7-H3 and B7x are highly expressed in human prostate cancer and associated with disease spread and poor outcome. Proc. Natl. Acad. Sci..

[71] Sica G.L., Choi I.H., Zhu G., Tamada K., Wang S.D., Tamura H., Chapoval A.I., Flies D.B., Bajorath J., Chen L. (2003). B7-H4, a Molecule of the B7 Family, Negatively Regulates T Cell Immunity. Immunity.

[72] Prasad D.V.R., Richards S., Mai X.M., Dong C. (2003). B7S1, a novel B7 family member that negatively regulates T cell activation. Immunity.

[73] Zang X., Loke P., Kim J., Murphy K., Waitz R., Allison J.P. (2003). B7x: A widely expressed B7 family member that inhibits T cell activation. Proc. Natl. Acad. Sci. USA.

[74] Jeon H., Vigdorovich V., Garrett-Thomson S.C., Janakiram M., Ramagopal U.A., Abadi Y.M., Lee J.S., Scandiuzzi L., Ohaegbulam K.C., Chinai J.M. (2014). Structure and cancer immunotherapy of the B7 family member B7x. Cell Rep..

[75] Dangaj D., Lanitis E., Zhao A., Joshi S., Cheng Y., Sandaltzopoulos R., Ra H.-J., Danet-Desnoyers G., Powell D.J.J., Scholler N. (2013). Novel recombinant human B7-H4 antibodies overcome tumoral immune escape to potentiate T-cell antitumor responses. Cancer Res..

[76] Li J., Lee Y., Li Y., Jiang Y., Lu H., Zang W., Zhao X., Liu L., Chen Y., Tan H. (2018). Co-inhibitory Molecule B7 Superfamily Member 1 Expressed by Tumor-Infiltrating Myeloid Cells Induces Dysfunction of Anti-tumor CD8(+) T Cells. Immunity.

[77] Nakanishi M., Rosenberg D.W. (2013). Multifaceted roles of PGE2 in inflammation and cancer. Semin. Immunopathol..

[78] Kalinski P. (2012). Regulation of immune responses by prostaglandin E2. J. Immunol..

[79] Goto T., Herberman R.B., Maluish A., Strong D.M. (1983). Cyclic AMP as a mediator of prostaglandin E-induced suppression of human natural killer cell activity. J. Immunol..

[80] Bankhurst A.D. (1982). The modulation of human natural killer cell activity by prostaglandins. J. Clin. Lab. Immunol..

[81] Specht C., Bexten S., Kölsch E., Pauels H.G. (2001). Prostaglandins, but not tumor-derived IL-10, shut down concomitant tumor-specific CTL responses during murine plasmacytoma progression. Int. J. Cancer.

[82] Obermajer N., Muthuswamy R., Lesnock J., Edwards R.P., Kalinski P. (2011). Positive feedback between PGE2 and COX2 redirects the differentiation of human dendritic cells toward stable myeloid-derived suppressor cells. Blood.

[83] Baratelli F., Lin Y., Zhu L., Yang S.-C., Heuzé-Vourc’h N., Zeng G., Reckamp K., Dohadwala M., Sharma S., Dubinett S.M. (2005). Prostaglandin E2 induces FOXP3 gene expression and T regulatory cell function in human CD4+ T cells. J. Immunol..

[84] Sharma S., Yang S.-C., Zhu L., Reckamp K., Gardner B., Baratelli F., Huang M., Batra R.K., Dubinett S.M. (2005). Tumor cyclooxygenase-2/prostaglandin E2-dependent promotion of FOXP3 expression and CD4+ CD25+ T regulatory cell activities in lung cancer. Cancer Res..

[85] Bergmann C., Strauss L., Zeidler R., Lang S., Whiteside T.L. (2007). Expansion of human T regulatory type 1 cells in the microenvironment of cyclooxygenase 2 overexpressing head and neck squamous cell carcinoma. Cancer Res..

[86] McLane L.M., Abdel-Hakeem M.S., Wherry E.J. (2019). CD8 T Cell Exhaustion During Chronic Viral Infection and Cancer. Annu. Rev. Immunol..

[87] Maraskovsky E., Chen W.F., Shortman K. (1989). IL-2 and IFN-gamma are two necessary lymphokines in the development of cytolytic T cells. J. Immunol..

[88] Curtsinger J.M., Agarwal P., Lins D.C., Mescher M.F. (2012). Autocrine IFN-γ promotes naive CD8 T cell differentiation and synergizes with IFN-α to stimulate strong function. J. Immunol..

[89] Duluc D., Corvaisier M., Blanchard S., Catala L., Descamps P., Gamelin E., Ponsoda S., Delneste Y., Hebbar M., Jeannin P. (2009). Interferon-gamma reverses the immunosuppressive and protumoral properties and prevents the generation of human tumor-associated macrophages. Int. J. Cancer.

[90] Caretto D., Katzman S.D., Villarino A.V., Gallo E., Abbas A.K. (2010). Cutting edge: The Th1 response inhibits the generation of peripheral regulatory T cells. J. Immunol..

[91] Olalekan S.A., Cao Y., Hamel K.M., Finnegan A. (2015). B cells expressing IFN-γ suppress Treg-cell differentiation and promote autoimmune experimental arthritis. Eur. J. Immunol..

[92] Liszewski M.K., Post T.W., Atkinson J.P. (1991). Membrane cofactor protein (MCP or CD46): Newest member of the regulators of complement activation gene cluster. Annu. Rev. Immunol..

[93] McDonald J.F., Nelsestuen G.L. (1997). Potent inhibition of terminal complement assembly by clusterin: Characterization of its impact on C9 polymerization. Biochemistry.

[94] Reis E.S., Mastellos D.C., Ricklin D., Mantovani A., Lambris J.D. (2018). Complement in cancer: Untangling an intricate relationship. Nat. Rev. Immunol..

[95] Timmermann B., Kerick M., Roehr C., Fischer A., Isau M., Boerno S.T., Wunderlich A., Barmeyer C., Seemann P., Koenig J. (2010). Somatic mutation profiles of MSI and MSS colorectal cancer identified by whole exome next generation sequencing and bioinformatics analysis. PLoS ONE.

[96] Zhao P., Li L., Jiang X., Li Q. (2019). Mismatch repair deficiency/microsatellite instability-high as a predictor for anti-PD-1/PD-L1 immunotherapy efficacy. J. Hematol. Oncol..

[97] Lizardo D.Y., Kuang C., Hao S., Yu J., Huang Y., Zhang L. (2020). Immunotherapy efficacy on mismatch repair-deficient colorectal cancer: From bench to bedside. Biochim. Biophys. Acta. Rev. Cancer.

[98] McGrail D.J., Pilié P.G., Rashid N.U., Voorwerk L., Slagter M., Kok M., Jonasch E., Khasraw M., Heimberger A.B., Lim B. (2021). High tumor mutation burden fails to predict immune checkpoint blockade response across all cancer types. Ann. Oncol. Off. J. Eur. Soc. Med. Oncol..

[99] Wang L., Pan S., Zhu B., Yu Z., Wang W. (2021). Comprehensive analysis of tumour mutational burden and its clinical significance in prostate cancer. BMC Urol..

[100] Gitto S.B., Kim H., Rafail S., Omran D.K., Medvedev S., Kinose Y., Rodriguez-Garcia A., Flowers A.J., Xu H., Schwartz L.E. (2020). An autologous humanized patient-derived-xenograft platform to evaluate immunotherapy in ovarian cancer. Gynecol. Oncol..

