# Supplementary material for: Framework of Intrinsic Immune Landscape of Dormant Prostate Cancer
Source: Cells. 2022 May 5;11(9):1550. doi: 10.3390/cells11091550 (PMC9105276; doi:10.3390/cells11091550)
Supplement: Supplementary file 1 [file cells-11-01550-s001.zip › Supplementary methods_20220425.pdf]

Supplementary methods

IHC staining of HLA-A

The processing and staining of the paraffin-embedded sections were performed as described in the Materials and Methods section 2.2. Anti-HLA-A antibody (manufacturer) was diluted at 1:250-500 for the application.

For quantification of immunohistochemistry, the staining intensity (In. 0, negative; 1, weak or incomplete membrane staining; 2, moderate intensity; 3, strong intensity) and the percentage of positive cells (P, ranging from 0-100%) was scored for each slide. The final score is calculated by the following formula: final score=In(1)×P(1)+ In(2)×P(2)+ In(3)×P(3).
