# Supplementary material for: Framework of Intrinsic Immune Landscape of Dormant Prostate Cancer
Source: Cells. 2022 May 5;11(9):1550. doi: 10.3390/cells11091550 (PMC9105276; doi:10.3390/cells11091550)
Supplement: Supplementary file 1 [file cells-11-01550-s001.zip › SupplementaryFigures_20220504.pptx]

## Slide 1
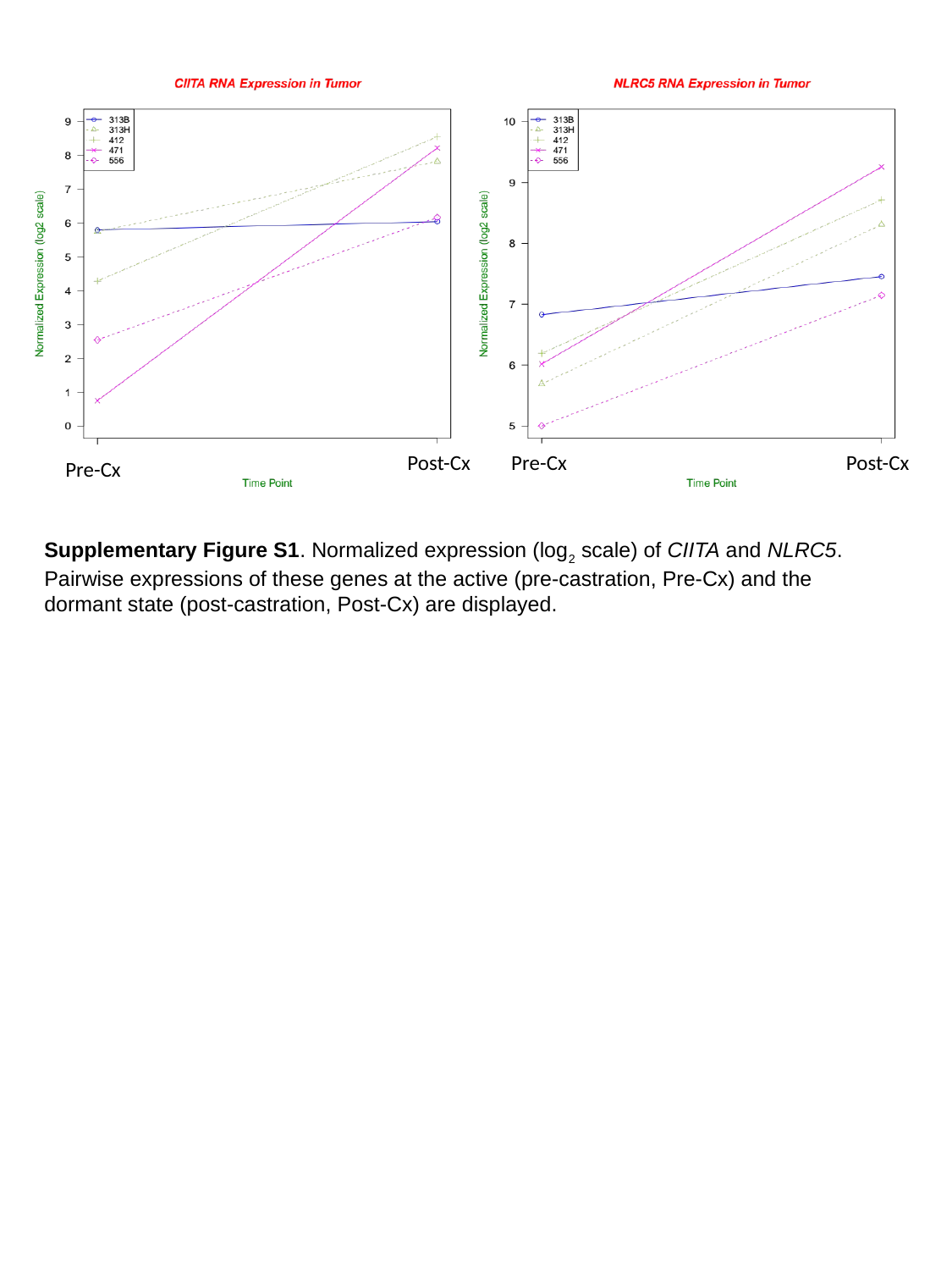

Post-Cx
Pre-Cx
Post-Cx
Pre-Cx
Supplementary Figure S1. Normalized expression (log2 scale) of CIITA and NLRC5. Pairwise expressions of these genes at the active (pre-castration, Pre-Cx) and the dormant state (post-castration, Post-Cx) are displayed.

## Slide 2
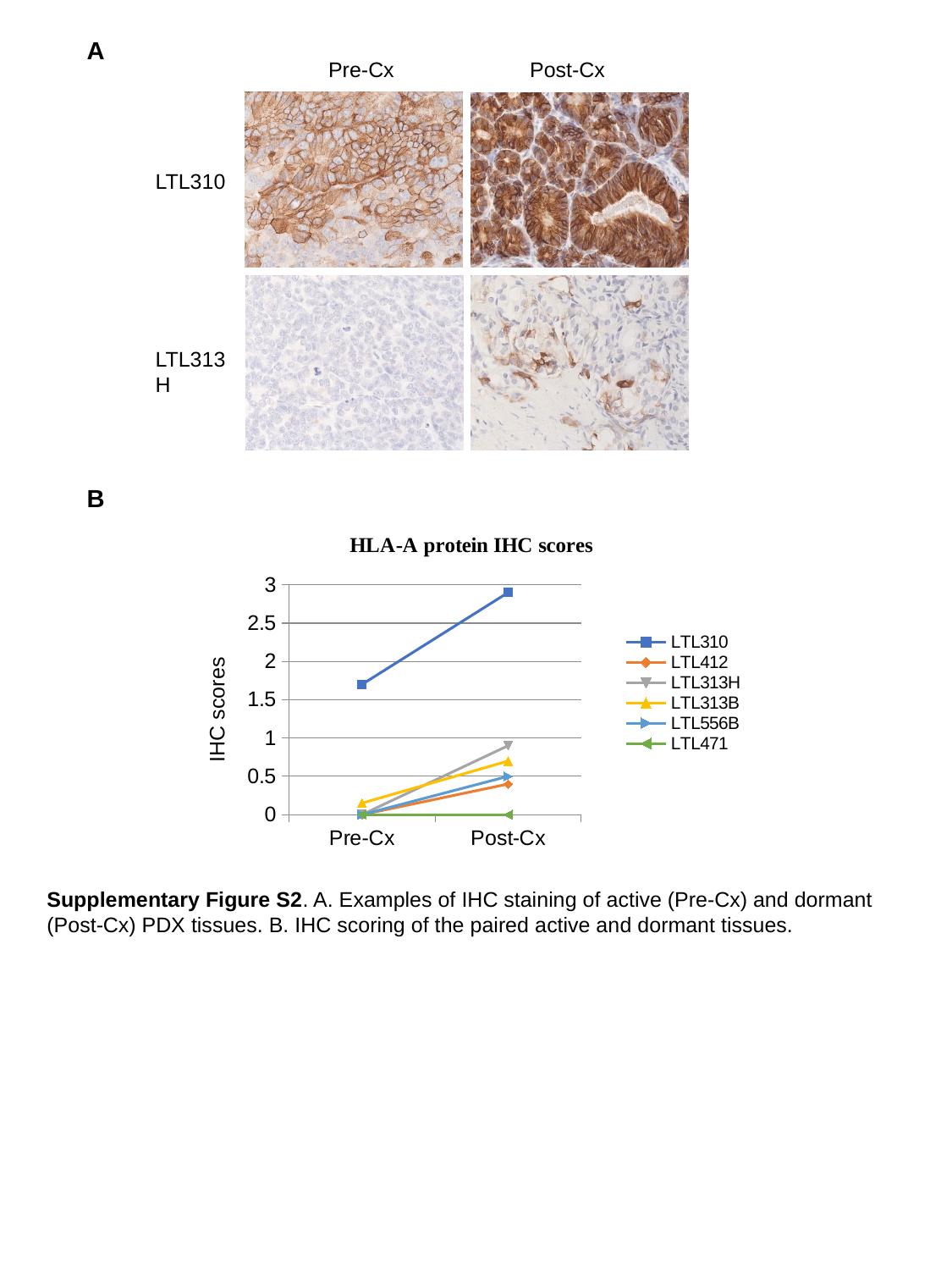

A
Pre-Cx Post-Cx
LTL310
LTL313H
B
### Chart
| Category | LTL310 | LTL412 | LTL313H | LTL313B | LTL556B | LTL471 |
|---|---|---|---|---|---|---|
| Pre-Cx | 1.700000000000001 | 0.0 | 0.0 | 0.15000000000000013 | 0.0 | 0.0 |
| Post-Cx | 2.9 | 0.4 | 0.9 | 0.7000000000000005 | 0.5 | 0.0 |IHC scores
Supplementary Figure S2. A. Examples of IHC staining of active (Pre-Cx) and dormant (Post-Cx) PDX tissues. B. IHC scoring of the paired active and dormant tissues.
